# A survey on fractal fractional nonlinear Kawahara equation theoretical and computational analysis

**DOI:** 10.1038/s41598-024-57389-0

**Published:** 2024-03-24

**Authors:** Laila A. Al-Essa, Mati ur Rahman

**Affiliations:** 1 Department of Mathematical Sciences, College of Science, Princess Nourah bint Abdulrahman University, P.O.Box 84428, 11671 Riyadh, Saudi Arabia; 2 School of Mathematical Sciences, Jiangsu University, Zhenjiang, 212013 Jiangsu People’s Republic of China; 3 Department of computer science and mathematics, Lebanese American university, Beirut , Lebanon

**Keywords:** Fractal-fractional operator, Kawahara equation, Fixed point, Unique solution, Applied mathematics, Computational science

## Abstract

With the use of the Caputo, Caputo-Fabrizio (CF), and Atangana-Baleanu-Caputo (ABC) fractal fractional differential operators, this study offers a theoretical and computational approach to solving the Kawahara problem by merging Laplace transform and Adomian decomposition approaches. We show the solution’s existence and uniqueness through generalized and advanced version of fixed point theorem. We present a precise and efficient method for solving nonlinear partial differential equations (PDEs), in particular the Kawahara problem. Through careful error analysis and comparison with precise solutions, the suggested method is validated, demonstrating its applicability in solving the nonlinear PDEs. Moreover, the comparative analysis is studied for the considered equation under the aforementioned operators.

## Introduction

Partial derivatives of an unknown function with respect to many variables are part of a partial differential equation (PDE), a particular kind of mathematical equation. In the realms of physics, engineering, and other disciplines, PDE’s are used to model a broad variety of events. They are frequently used to represent intricate systems that change over time and space, such as wave propagation, heat transfer, and fluid flow^1,2^. PDE’s can be categorised according to their order, linearity, and coefficients. The highest derivative in an equation determines the order of a PDE. For instance, the heat equation, a second-order PDE, defines how heat diffuses across a medium. A PDE’s linearity decides whether it is a linear or nonlinear equation^3^. Superposition techniques can be used to solve linear PDE’s, however numerical approaches are frequently needed to solve nonlinear PDE’s. PDE’s can be difficult to solve, hence numerous analytical and numerical techniques have been created to help. The separation of variables, the characteristic method, and Green’s functions are examples of analytical techniques. Finite element, boundary element, spectral, and finite difference approaches are examples of numerical techniques. In multiple branches of science and engineering, including heat transport, fluid mechanics, electromagnetic, and quantum physics, PDE’s are used. They are also utilised in biomedical engineering, finance, and image processing^4,5^.

In the 17th century, when the concept of fractional calculus (FC) first evolved, the mathematician Leibniz first wondered what would happen if the order of differentiation or integration was not a whole number. However, it wasn’t until the 20th century that FC began to develop as an independent subject^6–8^. One of the core notions of FC is the concept of fractional differential equations, fractional derivatives, and fractional integrals. We are able to define fractional derivatives by using fractional order operators, which are commonly denoted by the symbol D, where is a non-classical order^9–12^. Several issues, including the modeling of viscoelastic materials, the research of non-local mechanics, and the study of fractional diffusion processes, have been tackled via FC^13–16^. It is an extension of the integer order integrals and derivatives used in classical calculus. FC is used in many fields, including as physics, engineering, economics, and biology^17–20^.

The ideas of fractals and fractional calculus are joint to propose fractal fractional (FF) differential equations (DEs). Fractals are complex patterns produced at distinct sizes by self-similar geometric objects, while FC deals with derivatives and integrals of non-integer orders. FFDEs have recently attracted significant attention due to their ability to demonstrate complex phenomena that exhibit self-similarity at different scales, and their potential to give new insights into fundamental problems in physics and other fields. FF DEs are used in a variety of applied science subjects, including physics and mathematics^21–24^. FF DEs can be solved using analytical and numerical techniques like the spectral approach, integral transform techniques, and the finite difference method because they are often nonlinear in nature. The fractional Laplace equation, which extends the Laplace equation to non-integer order derivatives, is one of the most significant FFDEs. It is used to simulate diffusion in fractal media^25^. Another important FFDE is the fractional diffusion-wave equation, which is a generalization of the diffusion and wave equations to non-integer order derivatives. This equation is utilized to model wave propagation in fractal media and has uses in different subjects such as seismology, cosmology and mathematical physics^26^.

The KE is a nonlinear PDE that explain wave propagation in shallow water. It was initially given by Toshiaki Kawahara in 1978 as a model for wave propagation in a channel with slowly varying width^27^. The equation has since found uses in other areas, such as mathematical physics and fluid mechanics^28^. We consider the FF non-linear KE as

1 $$\begin{aligned} \frac{\partial ^{\sigma ,\,\vartheta } \omega }{\partial t^{\sigma }}-\frac{\partial ^{5} \omega }{\partial x^{5}}+\frac{\partial ^{3} \omega }{\partial x^{3}}+\omega \frac{\partial \omega }{\partial x}=0,\ \ \,0<\sigma ,\vartheta \le 1. \end{aligned}$$

Where, $\omega$ is the dependent variable, t is temporal, and x is spatial variables. and also$\sigma$ and $\vartheta$ denote the fractional and fractal orders, respectively. The first term on the left-hand side demonstrates the time evolution of $\omega$, while the other terms provide nonlinear and dispersive effects. Initial condition (IC) is given by

$$\begin{aligned} \omega (x,0)=f(x). \end{aligned}$$

The KE has been extensively analyzed in the literature, and many numerical and analytical techniques have been proposed to study its features. One interesting characteristic of the KE is the existence of solitary wave solutions^29^. Another interesting feature is the presence of instability regions in the parameter space, which can lead to the formation of chaotic patterns. The KE has been analyzed via various numerical and analytical methods, such as the kernel particle method^30^, the homotopy perturbation transform method^31–33^, the Galerkin procedure^34^, and the inverse scattering transform. These methods have been utilized to study the behavior of solitary waves, the stability features of the equation, and the formation of chaotic solutions.

In this work, we study the use of these nonlocal operators in solving the FF KE using the Laplace Adomain decomposition method (LADM). This method involves decomposing the equation into a set of simpler equations that can be handled analytically, followed by the inversion of the Laplace transform to figured out the solution in the original time domain. Additionlay, some qualitative features of FF KE are presented via fixed point theory. Our results illuminate the effectiveness of the Caputo, Caputo Fabrizio (CF), and Atangana-Baleanu (AB) fractional operators in accurately capturing the evolution of the system, and we show how our method can be used to analyze various physical phenomena, such as the propagation of waves in different domains.

## Basic definitions

Here, we elucidate some basic notions related to fractal-fractional (FF) calculus.

### **Definition 1**

Suppose $\omega \in H^1(p,~q)$, then the Caputo fractional operator (CFO) sense is

$$\begin{aligned} ^{C}_{a}D_t^{\sigma }\omega (t)=\frac{1}{\Gamma (m-\sigma )} \int _{a}^{t}(t-\varsigma )^{m-\sigma -1}\omega '(\varsigma )d\varsigma ,\,\,\,\,\,m-1<\sigma \le m. \end{aligned}$$

Suppose $\omega (t)$ is FF- differentiable in (*p*,  *q*), with fractal order $\vartheta$, then FF operator with power law kernel is

$$\begin{aligned} ^{FFP}_{a}D_t^{\sigma ,~\vartheta }\omega (t)=\frac{1}{\Gamma (m-\sigma )} \int _{a}^{t}(t-\varsigma )^{m-\sigma -1}\frac{d}{dt^{\vartheta }}\omega (\varsigma )d\varsigma ,\,\,\,\,\,0<m-1<\sigma ,~\vartheta \le m. \end{aligned}$$

### **Definition 2**

The FF integral with CFO is^35^

$$\begin{aligned} ^F_0 I_t^{\sigma , \vartheta }\omega (t)= \frac{\vartheta }{\Gamma (\sigma )}\int _{0}^{t}\varsigma ^{\sigma -1}\omega (\varsigma )(t-\varsigma )^{\sigma -1}d\varsigma . \end{aligned}$$

### **Definition 3**

Let $\omega (t)\in H(p,~q)$, $b > a$ and $\sigma \in [0,1]$, then the Caputo-Fabrizio fractional operator (CFFO) sense is

$$\begin{aligned} ^{CF}D^\sigma \omega (t)= \frac{A(\sigma )}{1-\sigma }\int _{a}^{t}\exp \left( \frac{-\sigma (t-\varsigma )}{1-\varsigma }\right) \omega (\varsigma )\,d\varsigma . \end{aligned}$$

### **Definition 4**

Let $\omega (t)$ is FF- differentiable. So the FF operator with CF operator having order $(\sigma ,\,\vartheta )$ of $\omega (t)$ is

$$\begin{aligned} ^{FFE}_{0}D_t^{\sigma ,\,\vartheta }\omega (t)=\frac{A(\sigma )}{1-\sigma } \int _{0}^{t} \exp \left( \frac{-\sigma (t-\varsigma )}{1-\varsigma }\right) \frac{d}{d\varsigma ^{\vartheta }}\omega (\varsigma )d\varsigma . \end{aligned}$$.

### **Definition 5**

Suppose that $\omega (t)\in H(p,~q)$, then the FF operator having ABC operator is

$$\begin{aligned} _{a}^{ABC}D_{t}^{\sigma }\omega (t)=\frac{A(\sigma )}{1-\sigma }\frac{d}{dt}\int _{a}^{t}E_{\sigma }\left( \frac{-\sigma (t-\varsigma )^{\sigma }}{1-\sigma }\right) \omega (\varsigma )d\varsigma ,\,\,\,\,\,\,n-1<\sigma \le n. \end{aligned}$$

### **Definition 6**

Let $\omega (t)$ is a fractal fractional differentiable, then the FF operator with ABC kernel is

$$\begin{aligned} ^{FFM}_{0}D_t^{\sigma ,\,\vartheta }\omega (t)=\frac{A(\sigma )}{1-\sigma } \int _{0}^{t} E_{\sigma }\left( \frac{-\sigma (t-\varsigma )^{\sigma }}{1-\sigma }\right) \frac{d}{d\varsigma ^{\vartheta }}\omega (\varsigma )d\varsigma . \end{aligned}$$

Where

$$\begin{aligned} A(\sigma )=1-\sigma +\frac{\sigma }{\Gamma (\sigma )}. \end{aligned}$$

### **Definition 7**

Laplace transform ${\textbf{L}}$, of a function $\omega (t)$, with $t>0$ as:

2 $$\begin{aligned} {\textbf{L}}[\omega (t)]=\int _{0}^{\infty }e^{-\varsigma t}\omega (\varsigma )d\varsigma . \end{aligned}$$

### **Definition 8**

If ${\textbf{L}}^{-1}\omega (\varsigma )=\omega (t)$, then ${\textbf{L}}^{-1}$ is:

$$\begin{aligned} {\textbf{L}}^{-1}\left( \frac{\omega (\varsigma )}{\varsigma }\right) =\int _{0}^{t}\omega (d)dt. \end{aligned}$$

### **Definition 9**

The Laplace transform (LT) of CFO as:

$$\begin{aligned} {\textbf{L}}[^{C}_{a}D_{t}^{\sigma }\omega (x,t)]=\varsigma ^{\sigma }{\textbf{L}}\omega (x,t)-\sum _{k=0}^{n-1}\varsigma ^{\sigma -k-1}\omega _{kt}(x,0), \,\,\,\, n=[\sigma ]+1. \end{aligned}$$

### **Definition 10**

The LT of CFFO is:

$$\begin{aligned} {\textbf{L}}[^{CF}_{0}D_{t}^{\sigma +m}\omega (x,t)]=\frac{A(\sigma )}{\varsigma +(1-\varsigma )\sigma }\left[ \varsigma ^{m}{\textbf{L}}\omega (x,t)-\sum _{k=0}^{m-1}\varsigma ^{m-k}\omega _{kx}(x,0)\right] . \end{aligned}$$

### **Definition 11**

The LT of ABC sense is:

$$\begin{aligned} {\textbf{L}}[^{ABC}_{0}D_{t}^{\sigma }\omega (x,t)]=\frac{A(\sigma )}{(1-\sigma )(\varsigma ^{\sigma }+\frac{\sigma }{(1-\sigma )})} \left[ \varsigma ^{m}{\textbf{L}}u(x,t)-\sum _{k=0}^{r}\varsigma ^{\sigma -r-k}\omega _{kx}(x,0)\right] . \end{aligned}$$

where $$r=[\sigma ]+1$$.

## Existence of the initial value problems

The existence and uniqueness of the initial value problems are studied in this section by using $\sigma$-type ${\mathfrak {F}}$-contraction^36^. For this purposes, Suppose $({\textbf{Z}}, d)$ be a complete metric space and $\varsigma$ be the family of strictly increasing functions ${\mathfrak {F}}:\mathfrak {R_{+}}\rightarrow {\mathfrak {R}}$ having the following properties:

- $\lim \limits _{n\rightarrow \infty }{\mathfrak {F}}(a_{n}) = -\infty$    if and only if, for each $\{a_{n}\}$, $\lim \limits _{n\rightarrow \infty }(a_{n})=0;$
- there exist $\upsilon \in (0,1)$ such that $\lim \limits _{a\rightarrow 0^{+}}a^{\upsilon }{\mathfrak {F}}(a)=0.$

### **Definition 12**

Let $\text{T}:{\textbf{Z}}\rightarrow {\textbf{Z}}$ be self mapping and $\sigma :{\textbf{Z}}\times {\textbf{Z}}\rightarrow [0,\infty )$, if

$$\begin{aligned} \sigma (\mathcal {Y},\mathcal {V}) \ge 1\Rightarrow & {} \sigma (\text{T}\mathcal {Y},\text{T}\mathcal {V})\ge 1, \end{aligned}$$

for all $\mathcal {Y},\mathcal {V}\in {\textbf{Z}}$, then $\text{T}$ is called $\sigma$-admissible.

### **Definition 13**

Let $({\textbf{Z}},d)$ be a complete metric space (CMS), $\text{T}:{\textbf{Z}}\rightarrow {\textbf{Z}}$ and $\sigma :{\textbf{Z}}\times {\textbf{Z}}\rightarrow \{-\infty \}\cup [0,\infty )$, there exist $\omega >0$ such that

$$\begin{aligned} \omega +\sigma (\text{Y},\mathcal {U}){\mathfrak {F}}(d(\text{T}\text{Y},\text{T}\mathcal {U}))\le & {} {\mathfrak {F}}(d(\text{Y},\mathcal {U})), \end{aligned}$$

for each $\text{Y},\mathcal {U}\in {\textbf{Z}}$ with $d(\text{T}\text{Y},\text{T}\mathcal {U})>0$. then $\text{T}$ is called $\sigma$-type ${\mathfrak {F}}$-contraction.

### **Theorem 1**

Let $({\textbf{Z}},d)$ be a CMS and $\text{T}:{\textbf{Z}}\rightarrow {\textbf{Z}}$ be an $\sigma$-type ${\mathfrak {F}}$-contraction such that

1. there exist $\text{Y}_{\circ }\in {\textbf{Z}}$ such that $\sigma (\text{Y}_{\circ },\text{T}\text{Y}_{\circ })\ge 1;$
2. if there exist $\{\text{Y}_{n}\}\subseteq {\textbf{Z}}$ with $\sigma (\text{Y}_{n},\text{Y}_{n+1})\ge 1$ and $\text{Y}_{n}\rightarrow \text{Y}$, then $\sigma (\text{Y}_{n},\text{Y})\ge 1$ for all $n\in \mathcal {P};$
3. $${\mathfrak {F}}$$ is continuous.

Then $\text{T}$ has a fixed point $\text{Y}^{*}\in {\textbf{Z}}$ also for $\text{Y}_{\circ }\in {\textbf{Z}}$ the sequence $\{\text{T}^{n}\text{Y}_{\circ }\}_{n\in \mathcal {P}}$ is convergent to $\text{Y}_{\circ }$

Let ${\textbf{Z}}=\mathcal {E}([0,1]^{2},{\mathfrak {R}})$ where $\mathcal {E}$ the space of all continuous functions $\text{Y}: [0,1]\times [0,1]\rightarrow {\mathfrak {R}}$ and $d(\text{Y}(x,t),\mathcal {U}(x,t))=\sup _{x,t\in [0,1]}[|\text{Y}(x,t)-\mathcal {U}(x,t)|]$, then we can write the IVP (1) in CF fractional derivative sense as:

3 $$\begin{aligned} ^{CF}D_t^\sigma \,\text{Y}(x,t)= & {} \vartheta t^{\vartheta -1}{\mathfrak {F}}(x,t,\text{Y}(x,t)),\, \,0<\sigma \le 1, \end{aligned}$$

with IC:

$$\begin{aligned} \text{Y}(x,0)=g(x),\, \, \end{aligned}$$

where $${\mathfrak {F}}(x,t,\text{Y}(x,t))=\omega _{xxxxx}-\omega _{xxx}-\omega \omega _{x}.$$

The theorem provided below gives the existence of solution of the problem (3)

### **Theorem 2**

There exist   ${\mathfrak {G}}:{\mathfrak {R}}^{2}\rightarrow {\mathfrak {R}}$ such that:

1. $$|{\mathfrak {F}}(x,t,\text{Y})-{\mathfrak {F}}(x,t,\mathcal {U})|\le \frac{(2-\sigma )A(\sigma )}{2} e^{b}|\text{Y}(x,t)-\mathcal {U}(x,t)|$$  for $$(x,t)\in [0,1]^{2}$$ and $$\text{Y},\mathcal {U}\in {\mathfrak {R}};$$
2. there exist $\text{Y}_{1}\in {\textbf{Z}}$ such that ${\mathfrak {G}}(\mathcal {Y_{1}},\text{T}\text{Y}_{1})\ge 0$, where $\text{T}:{\textbf{Z}}\rightarrow {\textbf{Z}}$ defined by
  $$\begin{aligned} \text{T}\text{Y}=\text{Y}_{\circ }+\vartheta t^{\vartheta -1} {}^{CF}_{0}I^{\sigma }{\mathfrak {F}}(x,t,\text{Y}(x,t)); \end{aligned}$$
3. for $\text{Y},\mathcal {U}\in {\textbf{Z}}, {\mathfrak {G}}(\text{Y},\mathcal {U})\ge 0$ implies that ${\mathfrak {G}}(\text{T}\text{Y},\text{T}\mathcal {U})\ge 0$;
4. $\{\text{Y}_{n}\}\subseteq {\textbf{Z}}, \lim \limits _{n\rightarrow \infty }\text{Y}_{n}=\text{Y}$, where $\text{Y}\in {\textbf{Z}}$ and ${\mathfrak {G}}(\text{Y}_{n},\text{Y}_{n+1})\ge 0$ implies that ${\mathfrak {G}}(\text{Y}_{n},\text{Y})\ge 0,$ for all $n\in \mathcal {P}$

Then there exist at least one fixed point of $\text{T}$ which is the solution of the problem (3).

### *Proof*

To prove that $\text{T}$ has a fixed point, therefore

$$\begin{aligned}{} & {} |\text{T}\text{Y}-\text{T}\mathcal {U}||\text{T}\text{Y}-\text{T}\mathcal {U}+1|\\{} & {} \quad =|\vartheta t^{\vartheta -1} {}^{CF}I[{\mathfrak {F}}(x,t\text{Y})-{\mathfrak {F}}(x,t\mathcal {U})]||\vartheta t^{\vartheta -1} {}^{CF}I[{\mathfrak {F}}(x,t\text{Y})-{\mathfrak {F}}(x,t\mathcal {U})]+1| \\{} & {} \quad \le (\vartheta T^{\vartheta -1})\big (\frac{2(1-\sigma )}{(2-\sigma )A(\sigma )}|{\mathfrak {F}}(x,t\text{Y})-{\mathfrak {F}}(x,t\mathcal {U})|\\{} & {} \quad \quad +\frac{2 \sigma }{(2-\sigma )A(\sigma )}\int _{0}^{\tau }|{\mathfrak {F}}(x,t\text{Y})-{\mathfrak {F}}(x,t\mathcal {U})|d\tau \big )\\{} & {} \quad \quad \times (\vartheta T^{\vartheta -1})\big (\frac{2(1-\sigma )}{(2-\sigma )A(\sigma )}|{\mathfrak {F}}(x,t\text{Y})-{\mathfrak {F}}(x,t\mathcal {U})|\\{} & {} \quad \quad +\frac{2 \sigma }{(2-\sigma )A(\sigma )}\int _{0}^{\tau }|{\mathfrak {F}}(x,t\text{Y})-{\mathfrak {F}}(x,t\mathcal {U})|d\tau +1\big ) \\{} & {} \quad \le (\vartheta T^{\vartheta -1})\big (\frac{2(1-\sigma )}{(2-\sigma )A(\sigma )}.\frac{(2-\sigma )A(\sigma )}{2} e^{-b}|\text{Y}-\mathcal {U}|\\{} & {} \quad \quad +\frac{2\sigma }{(2-\sigma )A(\sigma )}.\frac{(2-\sigma )A(\sigma )}{2}\int _{0}^{\tau }e^{-b}|\text{Y}-\mathcal {U}|d\tau \big )\\{} & {} \quad \quad \times (\vartheta T^{\vartheta -1})\big (\frac{2(1-\sigma )}{(2-\sigma )A(\sigma )}.\frac{(2-\sigma )A(\sigma )}{2}e^{-b}|\text{Y}-\mathcal {U}|\\{} & {} \quad \quad +\frac{2(1-\sigma )}{(2-\sigma )A(\sigma )}.\frac{(2-\sigma )A(\sigma )}{2}\int _{0}^{\tau }e^{-b}|\text{Y}-\mathcal {U}|d\tau +1\big ) \\{} & {} \quad \le (\vartheta T^{\vartheta -1}) \big (e^{-b}\sup _{x,t\in [0,1]}|\text{Y}(x,t)-\mathcal {U}(x,t)|\big )\big (e^{-b}\sup _{x,t\in [0,1]}|\text{Y}(x,t)-\mathcal {U}(x,t)|+1\big ) \\{} & {} \quad = (\vartheta T^{\vartheta -1})\big ( e^{-b}d(\text{Y},\mathcal {U})\big )\big (e^{-b}d(\text{Y},\mathcal {U})+1\big )\\{} & {} \quad = (\vartheta T^{\vartheta -1})e^{-b}[e^{-b}(d(\text{Y},\mathcal {U}))^{2}+d(\text{Y},\mathcal {U})] \\{} & {} \quad \le (\vartheta T^{\vartheta -1})e^{-b}[(d(\text{Y},\mathcal {U}))^{2}+d(\text{Y},\mathcal {U})]. \end{aligned}$$

Thus for $\text{Y},\mathcal {U}\in {\textbf{Z}}$ with ${\mathfrak {G}}(\text{Y},\mathcal {U})\ge 0,$ we obtain

$$\begin{aligned}{} & {} (d(\text{T}\text{Y},\text{T}\mathcal {U}))^{2}+d(\text{T}\text{Y},\text{T}\mathcal {U})\\{} & {} \quad \le (\vartheta T^{\vartheta -1})e^{-b}[(d(\text{Y},\mathcal {U}))^{2}+d(\text{Y},\mathcal {U})]. \end{aligned}$$

Taking $\ln$ on both sides, we have:

$$\begin{aligned} b+\ln [d(\text{T}\text{Y},\text{T}\mathcal {U}))^{2}+d(\text{T}\text{Y},\text{T}\mathcal {U}]\le & {} (\vartheta T^{\vartheta -1})\ln [d(\text{Y},\mathcal {U}))^{2}+d(\text{Y},\mathcal {U}] \end{aligned}$$

if ${\mathfrak {F}}:[0,\infty )\rightarrow {\mathfrak {R}}$ defined by ${\mathfrak {F}}({\mathfrak {u}})=(\vartheta T^{\vartheta -1})\ln [{\mathfrak {u}}^{2}+{\mathfrak {u}}],~~{\mathfrak {u}}>0$, then ${\mathfrak {F}}\in \delta$.

Now define $\sigma :{\textbf{Z}}\times {\textbf{Z}}\rightarrow \{-\infty \}\cup [0,\infty )$ as

$$\begin{aligned} \sigma (\text{Y},\mathcal {U})= & {} {\left\{ \begin{array}{ll} 1, &{} \text{ if } {\mathfrak {G}}(\text{Y}(x,t),\mathcal {U}(x,t))\ge 0 ~~for~~ all~~ x,t\in [0,1] \\ -\infty , &{} \text{ otherwise }. \end{array}\right. } \end{aligned}$$

Then

$$\begin{aligned} b+\sigma (\text{Y},\mathcal {U}){\mathfrak {F}}\big (d(\text{T}\text{Y},\text{T}\mathcal {U})\big )\le & {} {\mathfrak {F}}(d(\text{Y},\mathcal {U})) \end{aligned}$$

for $\text{Y},\mathcal {U}\in {\textbf{Z}}$ with $d(\text{T}\text{Y},\text{T}\mathcal {U})>0$. Now by ${\mathfrak {G}}3,$

$$\begin{aligned} \sigma (\text{Y},\mathcal {U}) \ge 1~~\Rightarrow & {} ~~{\mathfrak {G}}(\text{Y},\mathcal {U})\ge 0~~\Rightarrow ~~{\mathfrak {G}}(\text{T}\text{Y},\text{T}\mathcal {U})\ge 0\\\Rightarrow & {} \sigma (\text{T}\text{Y},\text{T}\mathcal {U})\ge 1 \end{aligned}$$

for all $\text{Y},\mathcal {U}\in {\textbf{Z}}$. From ${\mathfrak {G}}2$ there exist $\text{Y}_{\circ }\in {\textbf{Z}}$ such that $\sigma (\text{Y}_{\circ },\text{T}\text{Y}_{\circ })\ge 1.$ therefore by ${\mathfrak {G}}4$ and Theorem 1, there exist $\text{Y}^{*}\in {\textbf{Z}}$ such that $\text{Y}^{*}=\text{T}\text{Y}^{*}$. Hence $\mathcal {Y}^{*}$ is the solution of the problem (3) $\square$

Similarly we can write the IVP (1) in ABC sense as

4 $$\begin{aligned} ^{ABC}D_t^{\sigma ,\vartheta }\,\text{Y}(x,t)= & {} \vartheta t^{\vartheta -1}{\mathfrak {F}}(x,t,\text{Y}(x,t)),\, \,0<\sigma \le 1, \end{aligned}$$

with IC

$$\begin{aligned} \text{Y}(x,0)=g(x),\, \, \end{aligned}$$

where $${\mathfrak {F}}(x,t,\text{Y}(x,t))=\omega _{xxxxx}-\omega _{xxx}-\omega \omega _{x}.$$

The following theorem show the existence of solution of the problem (4)

### **Theorem 3**

There exist   ${\mathfrak {G}}:{\mathfrak {R}}^{2}\rightarrow {\mathfrak {R}}$ such that

1. $$|{\mathfrak {F}}(x,t,\text{Y})-{\mathfrak {F}}(x,t,\mathcal {U})|\le \frac{(\Gamma \sigma ) \sigma }{(1-\sigma )\Gamma (\sigma +1)} e^{\frac{-b}{2}}|\text{Y}(x,t)-\mathcal {U}(x,t)|$$  for $$(x,t)\in [01]^{2}$$ and $$\text{Y},\mathcal {U}\in {\mathfrak {R}};$$
2. there exist $\text{Y}_{1}\in {\textbf{Z}}$ such that ${\mathfrak {G}}(\mathcal {Y_{1}},\text{T}\text{Y}_{1})\ge 0$, where $\text{T}:{\textbf{Z}}\rightarrow {\textbf{Z}}$ defined by
  $$\begin{aligned} \text{T}\text{Y}=\text{Y}_{\circ }+{}^{ABC}_{0}I^{\sigma }\vartheta t^{\vartheta -1}{\mathfrak {F}}(x,t,\text{Y}(x,t)); \end{aligned}$$
3. for $\text{Y},\mathcal {U}\in {\textbf{Z}}, {\mathfrak {G}}(\text{Y},\mathcal {U})\ge 0$ implies that ${\mathfrak {G}}(\text{T}\text{Y},\text{T}\mathcal {U})\ge 0$;
4. $\{\text{Y}_{n}\}\subseteq {\textbf{Z}}, \lim \limits _{n\rightarrow \infty }\text{Y}_{n}=\text{Y}$, where $\text{Y}\in {\textbf{Z}}$ and ${\mathfrak {G}}(\text{Y}_{n},\text{Y}_{n+1})\ge 0$ implies that ${\mathfrak {G}}(\text{Y}_{n},\text{Y})\ge 0,$ for all $n\in \mathcal {P}$.

Then there exist at least one fixed point of $\text{T}$ which is the solution of the problem (3).

### *Proof*

 

$$\begin{aligned} |\text{T}\text{Y}-\text{T}\mathcal {U}|^{2}= & {} |{}^{AB}_{0}I^{\sigma }(\vartheta t^{\vartheta -1})[{\mathfrak {F}}(x,t,\text{Y}(x,t))-{\mathfrak {F}}(x,t,\mathcal {U}(x,t))]|^{2} \\\le & {} (\vartheta T^{\vartheta -1})\bigg [|\frac{1-\sigma }{A(\sigma )}[{\mathfrak {F}}(x,t,\text{Y})-{\mathfrak {F}}(x,t,\mathcal {U})]+ \frac{\sigma }{A(\sigma )}{}_{0}I^{\sigma }[\mathcal {F}(x,t,\mathcal {Y}(x,t))-\mathcal {F}(x,t,\mathcal {V}(x,t))]|\bigg ]^{2}\\\le & {} (\vartheta T^{\vartheta -1})\bigg [\frac{1-\sigma }{A(\sigma )}|{\mathfrak {F}}(x,t,\text{Y})-{\mathfrak {F}}(x,t,\mathcal {U})| +\frac{\sigma }{A(\sigma )}{}_{0}I^{\sigma }|\mathcal {F}(x,t,\mathcal {Y}(x,t))-\mathcal {F}(x,t,\mathcal {V}(x,t))|\bigg ]^{2}\\\le & {} (\vartheta T^{\vartheta -1}) \bigg [\frac{1-\sigma }{A(\sigma )}.\frac{A(\sigma )(\Gamma \sigma ) }{(1-\sigma )\Gamma (\sigma +1)}e^{\frac{-b}{2}}\sqrt{|\mathcal {Y}-\mathcal {V}|^{2}}\\{} & {} +\frac{\sigma }{A(\sigma )}\frac{A(\sigma )(\Gamma \sigma ) }{(1-\sigma )\Gamma (\sigma +1)}{}_{0}I^{\sigma }1.e^{\frac{-b}{2}}\sqrt{|\mathcal {Y}-\mathcal {V}|^{2}}\bigg ]^{2}\\= & {} (\vartheta T^{\vartheta -1}) \bigg [\frac{A(\sigma )(\Gamma \sigma ) }{(1-\sigma )\Gamma (\sigma +1)}e^{\frac{-b}{2}}\sqrt{|\mathcal {Y}-\mathcal {V}|^{2}}\}^{2}\\{} & {} \{\frac{1-\sigma }{A(\sigma )}+\frac{\sigma }{A(\sigma )\sigma (\Gamma \sigma ) }\bigg ]^{2}\\\le & {} (\vartheta T^{\vartheta -1})\bigg [\frac{A(\sigma )(\Gamma \sigma ) }{(1-\sigma )\Gamma (\sigma +1)}e^{\frac{-b}{2}}\sqrt{\sup _{x,t\in [0,1]}|\mathcal {Y}(x,t)-\mathcal {V}(x,t)|^{2}}\bigg ]^{2}\\{} & {} \bigg [\frac{1-\sigma }{A(\sigma )}+\frac{\sigma }{A(\sigma )\sigma (\Gamma \sigma )}\bigg ]^{2}\\= & {} (\vartheta T^{\vartheta -1})\bigg [\frac{A(\sigma )(\Gamma \sigma )}{(1-\sigma )\Gamma (\sigma +1)}e^{\frac{-b}{2}}\sqrt{d(\mathcal {Y},\mathcal {V})}\bigg ]^{2}\\{} & {} \bigg [\frac{1-\sigma }{A(\sigma )}+\frac{\sigma }{A(\sigma )(\Gamma \sigma )}\bigg ]^{2}\\= & {} (\vartheta T^{\vartheta -1}) e^{-b}d(\mathcal {Y},\mathcal {V}). \end{aligned}$$

Consequently

$$\begin{aligned} d(\text{T}\mathcal {Y},\text{T}\mathcal {V})\le & {} (\vartheta T^{\vartheta -1})e^{-b}d(\mathcal {Y},\mathcal {V}). \end{aligned}$$

Applying $``\ln ''$ which implies that

$$\begin{aligned} \ln (d(\text{T}\mathcal {Y},\text{T}\mathcal {V}))\le (\vartheta T^{\vartheta -1})\ln (e^{-b}d(\mathcal {Y},\mathcal {V})), \end{aligned}$$

and

$$\begin{aligned} b+\ln (d(\text{T}\mathcal {Y},\text{T}\mathcal {V})\le (\vartheta T^{\vartheta -1})\ln (d(\mathcal {Y},\mathcal {V})). \end{aligned}$$

Let ${\mathfrak {F}}:[0,\infty )\rightarrow {\mathfrak {R}}$ define by ${\mathfrak {F}}(\lambda )=\ln \lambda ,$ where $\lambda >0$, then it is easy to show that ${\mathfrak {F}}\in \varsigma$.

Now define $\sigma :{\textbf{Z}}\times {\textbf{Z}}\rightarrow \{-\infty \}\cup [0,\infty )$ by

$$\begin{aligned} \sigma (\mathcal {Y},\mathcal {V})={\left\{ \begin{array}{ll} 1, &{} \text{ if } {\mathfrak {G}}(\mathcal {Y}(x,t),\mathcal {V}(x,t))\ge 0~~for~all~x,t \in [0,1] \\ -\infty , &{} \text{ otherwise }. \end{array}\right. } \end{aligned}$$

Thus $b+\sigma (\mathcal {Y},\mathcal {V}){\mathfrak {F}}(d(\text{T}\mathcal {Y},\text{T}\mathcal {V}))\le {\mathfrak {F}}(d(\mathcal {Y},\mathcal {V}))$ for $\mathcal {Y},\mathcal {V}\in {\textbf{Z}}$ with $d(\text{T}\mathcal {Y},\text{T}\mathcal {V})\ge 0$. therefore $\text{T}$ is an $\sigma$-type ${\mathfrak {F}}$-contraction. From $({\mathfrak {G}}3)$ we have

$$\begin{aligned} \sigma (\mathcal {Y},\mathcal {V})\ge 1\Rightarrow & {} {\mathfrak {G}}(\mathcal {Y},\mathcal {V})\ge 0~~\Rightarrow ~~{\mathfrak {G}}(\text{T}\mathcal {Y},\text{T}\mathcal {V}),\\\Rightarrow & {} \sigma (\text{T}\mathcal {Y},\text{T}\mathcal {V})\ge 1, \end{aligned}$$

for all $x,t\in [0,1]$. Thus $\text{T}$ is an $\sigma$-admissible. From $({\mathfrak {G}}2)$ there exist $\mathcal {Y}_{\circ }\in {\textbf{Z}}$ with $\sigma (\mathcal {Y}_{\circ },\text{T}\mathcal {Y}_{\circ })\ge 1.$ From $({\mathfrak {G}}4)$, there exist $\mathcal {Y}^{*}\in {\textbf{Z}}$ such that $\text{T}\mathcal {Y}^{*}$. Hence $\mathcal {Y}^{*}$ is the solution of the initial value problem (4). $\square$

## Proposed method

Here, we develop the Laplace transform of the FF operators with different kernels. Next, we’ll use LADM to roughly solve the system under consideration.

### Scheme for the proposed model with CFO

Equation (1) in terms of the Caputo operator, which is provided by.

5 $$\begin{aligned} ^{C}D_t^{\sigma ,\,\vartheta }\omega -\frac{\partial ^{5} \omega }{\partial x^{5}}+\frac{\partial ^{3} \omega }{\partial x^{3}}+\omega \frac{\partial \omega }{\partial x}=0,\ \ \,0<\sigma ,\vartheta \le 1, \end{aligned}$$

with IC,

6 $$\begin{aligned} \omega (x,0)=f(x). \end{aligned}$$

Equivalent form of Eq. (5) is:

7 $$\begin{aligned} ^{C}D_t^{\sigma }\omega= & {} \vartheta t^{\vartheta -1}\bigg [\frac{\partial ^{5} \omega }{\partial x^{5}}-\frac{\partial ^{3} \omega }{\partial x^{3}}-\omega \frac{\partial \omega }{\partial x}\bigg ]. \end{aligned}$$

Applying LT to Eq. (7), we get:

8 $$\begin{aligned} {\textbf{L}}[^{C}D_t^{\sigma }\omega ]= & {} {\textbf{L}}\bigg [\vartheta t^{\vartheta -1}\{\frac{\partial ^{5} \omega }{\partial x^{5}}-\frac{\partial ^{3} \omega }{\partial x^{3}}-\omega \frac{\partial \omega }{\partial x}\}\bigg ]. \end{aligned}$$

In “Basic definitions” section on the power law kernel, we discussed the definition of the LT.

9 $$\begin{aligned} {\textbf{L}}[^{C}D_t^{\sigma }\omega ]= & {} \frac{q(x)}{s}+\frac{1}{s^{\sigma }}{\textbf{L}}\left[ \vartheta t^{\vartheta -1}(\frac{\partial ^{5} \omega }{\partial x^{5}}-\frac{\partial ^{3} \omega }{\partial x^{3}}-\omega \frac{\partial \omega }{\partial x} \right] . \end{aligned}$$

The series solution can be expressed as:

$$\begin{aligned} \omega (x,t)=\sum _{n=0}^\infty \omega _n(x,t). \end{aligned}$$

The decomposed non-linear terms are as follows:

$$\begin{aligned} \omega (x,t)=\sum _{i=0}^\infty E_n, \end{aligned}$$

where $E_n$ denotes Adomian polynomials $\omega _0,\omega _1,\omega _2, \dots$,

10 $$\begin{aligned} E_n=\frac{1}{n!}\frac{d^n}{d\lambda ^n}\left[ \sum _{k=0}^n \lambda ^k\omega _k(x,t)\right] _{\lambda =0}. \end{aligned}$$

Applying ${\textbf{L}}^{-1}$ to Eq. (9) together with Eq. (10), we get:

11 $$\begin{aligned} \sum _{n=0}^{\infty }\omega _{n}(x,t)=f(x))+{\textbf{L}}^{-1}\left[ \frac{1}{s^{\sigma }}{\textbf{L}}\left\{ \vartheta t^{\vartheta -1}\left( \sum _{n=0}^{\infty }\omega _{nxxxxx}-\sum _{n=0}^{\infty }\omega _{nxxx}-\sum _{n=0}^{\infty }E_n\right) \right\} \right] . \end{aligned}$$

The series solution is obtained by comparing the terms on both sides of Eq. (11).

$$\begin{aligned} \omega _{0}= &\,  \omega (x,0),\\ \omega _{1}= &\, {\textbf{L}}^{-1}\left[ \frac{1}{s^{\sigma }}{\textbf{L}}\left\{ \vartheta t^{\vartheta -1}(\omega _{0xxxxx}-\omega _{0xxx}-E_{0})\right\} \right] ,\\ \omega _{2}= &\,{\textbf{L}}^{-1}\left[ \frac{1}{s^{\sigma }}{\textbf{L}}\left\{ \vartheta t^{\vartheta -1}(\omega _{1xxxxx}-\omega _{1xxx}-E_{1})\right\} \right] ,\\ \omega _{3}= &\, {\textbf{L}}^{-1}\left[ \frac{1}{s^{\sigma }}{\textbf{L}}\left\{ \vartheta t^{\vartheta -1}(\omega _{2xxxxx}-\omega _{2xxx}-E_{2})\right\} \right] ,\\ \vdots . \end{aligned}$$

The series can written as:     $\omega (x,t)=\sum _{n=0}^\infty \omega _n(x,t)$.

### Scheme for the proposed model with CFFO

12 $$\begin{aligned} ^{{FFE}}D_t^{\sigma ,\,\vartheta }\omega -\frac{\partial ^{5} \omega }{\partial x^{5}}+\frac{\partial ^{3} \omega }{\partial x^{3}}+\omega \frac{\partial \omega }{\partial x}=0,\ \ \,0<\sigma ,\vartheta \le 1, \end{aligned}$$

with IC,

13 $$\begin{aligned} \omega (x,0)=f(x). \end{aligned}$$

Equivalently Eq. (12) gets the form:

14 $$\begin{aligned} ^{FFE}D_t^{\sigma }\omega= & {} \vartheta t^{\vartheta -1}\bigg [\frac{\partial ^{5} \omega }{\partial x^{5}}-\frac{\partial ^{3} \omega }{\partial x^{3}}-\omega \frac{\partial \omega }{\partial x}\bigg ]. \end{aligned}$$

Applying LT to Eq. (14), we get:

15 $$\begin{aligned} {\textbf{L}}[^{FFE}D_t^{\sigma }\omega ]= & {} {\textbf{L}}[\vartheta t^{\vartheta -1}\{\frac{\partial ^{5} \omega }{\partial x^{5}}-\frac{\partial ^{3} \omega }{\partial x^{3}}-\omega \frac{\partial \omega }{\partial x}\}]. \end{aligned}$$

In “Basic definitions” section on the exponential decay kernel, we addressed the definition of the LT.

16 $$\begin{aligned} {\textbf{L}}[^{{FFE}}D_t^{\sigma }\omega ]= & {} \frac{K(x)}{s}+\frac{(s+( 1-s)\sigma )}{s}{\textbf{L}}\left[ \vartheta t^{\vartheta -1}(\frac{\partial ^{5} \omega }{\partial x^{5}}-\frac{\partial ^{3} \omega }{\partial x^{3}}-\omega \frac{\partial \omega }{\partial x} \right] . \end{aligned}$$

The whole series solution can be scripted as,

$$\begin{aligned} \omega (x,t)=\sum _{n=0}^\infty \omega _n(x,t). \end{aligned}$$

the non-linear terms are decomposed with Adomian-polynomial discussed above.

Applying ${\textbf{L}}^{-1}$ to Eq. (16), we get

17 $$\begin{aligned} \sum _{n=0}^{\infty }\omega _{n}(x,t)=f(x))+{\textbf{L}}^{-1}\left[ \left( 1-\sigma +\frac{\sigma }{s}\right) {\textbf{L}}\left\{ \vartheta t^{\vartheta -1}\left( \sum _{n=0}^{\infty }\omega _{nxxxxx}-\sum _{n=0}^{\infty }\omega _{nxxx}-\sum _{n=0}^{\infty }E_n\right) \right\} \right] . \end{aligned}$$

Equating terms on both sides in Eq. (17), we get:

$$\begin{aligned} \omega _{0}= &\, \omega (x,0),\\ \omega _{1}= &\, {\textbf{L}}^{-1}\left[ \left( 1-\sigma +\frac{\sigma }{s}\right) {\textbf{L}}\left\{ \vartheta t^{\vartheta -1}(\omega _{0xxxxx}-\omega _{0xxx}-E_{0})\right\} \right] ,\\ \omega _{2}= &\, {\textbf{L}}^{-1}\left[ \left( 1-\sigma +\frac{\sigma }{s}\right) {\textbf{L}}\left\{ \vartheta t^{\vartheta -1}(\omega _{1xxxxx}-\omega _{1xxx}-E_{1})\right\} \right] ,\\ \omega _{3}= &\, {\textbf{L}}^{-1}\left[ \left( 1-\sigma +\frac{\sigma }{s}\right) {\textbf{L}}\left\{ \vartheta t^{\vartheta -1}(\omega _{2xxxxx}-\omega _{2xxx}-E_{2})\right\} \right] ,\\ \vdots . \end{aligned}$$

The series can written as:     $\omega (x,t)=\sum _{n=0}^\infty \omega _n(x,t)$.

### Scheme for the proposed model with ABC operator

18 $$\begin{aligned} ^{FFM}D_t^{\sigma ,\,\vartheta }\omega -\frac{\partial ^{5} \omega }{\partial x^{5}}+\frac{\partial ^{3} \omega }{\partial x^{3}}+\omega \frac{\partial \omega }{\partial x}=0,\ \ \,0<\sigma ,\vartheta \le 1, \end{aligned}$$

with IC:

19 $$\begin{aligned} \omega (x,0)=f(x). \end{aligned}$$

Equivalently Eq. (18) can written as:

20 $$\begin{aligned} ^{FFM}D_t^{\sigma }\omega= & {} \vartheta t^{\vartheta -1}\bigg [\frac{\partial ^{5} \omega }{\partial x^{5}}-\frac{\partial ^{3} \omega }{\partial x^{3}}-\omega \frac{\partial \omega }{\partial x}\bigg ]. \end{aligned}$$

Applying LT to Eq. (20), we obtain:

21 $$\begin{aligned} {\textbf{L}}[^{FFM}D_t^{\sigma }\omega ]= & {} {\textbf{L}}[\vartheta t^{\vartheta -1}\{\frac{\partial ^{5} \omega }{\partial x^{5}}-\frac{\partial ^{3} \omega }{\partial x^{3}}-\omega \frac{\partial \omega }{\partial x}\}]. \end{aligned}$$

Using the definition of LT discussed in “Basic definitions” section on Mittag-Leffler kernel, we get:

22 $$\begin{aligned} {\textbf{L}}[^{FFM}D_t^{\sigma }\omega ]= & {} \frac{K(x)}{s}+\left( (1-\sigma )+\frac{\sigma }{s^{\sigma }}\right) {\textbf{L}}\left[ \vartheta t^{\vartheta -1}(\frac{\partial ^{5} \omega }{\partial x^{5}}-\frac{\partial ^{3} \omega }{\partial x^{3}}-\omega \frac{\partial \omega }{\partial x}) \right] . \end{aligned}$$

The whole series solution can be scripted as:

$$\begin{aligned} \omega (x,t)=\sum _{n=0}^\infty \omega _n(x,t). \end{aligned}$$

the non-linear terms are decomposed with Adomian-polynomial discussed above. Applying ${\textbf{L}}^{-1}$ to Eq. (22), we get

23 $$\begin{aligned} \sum _{n=0}^{\infty }\omega _{n}(x,t)=f(x))+{\textbf{L}}^{-1}\left[ \left( (1-\sigma )+\frac{\sigma }{s^{\sigma }}\right) {\textbf{L}}\left\{ \vartheta t^{\vartheta -1}\left( \sum _{n=0}^{\infty }\omega _{nxxxxx}-\sum _{n=0}^{\infty }\omega _{nxxx}-\sum _{n=0}^{\infty }E_n\right) \right\} \right] . \end{aligned}$$

Equating terms on both sides in Eq. (23), we get:

$$\begin{aligned} \omega _{0}= &\, \omega (x,0),\\ \omega _{1}= & {} {\textbf{L}}^{-1}\left[ \left( (1-\sigma )+\frac{\sigma }{s^{\sigma }}\right) {\textbf{L}}\left\{ \vartheta t^{\vartheta -1}(\omega _{0xxxxx}-\omega _{0xxx}-E_{0})\right\} \right] ,\\ \omega _{2}= &\, {\textbf{L}}^{-1}\left[ \left( (1-\sigma )+\frac{\sigma }{s^{\sigma }}\right) {\textbf{L}}\left\{ \vartheta t^{\vartheta -1}(\omega _{1xxxxx}-\omega _{1xxx}-E_{1})\right\} \right] ,\\ \omega _{3}= &\, {\textbf{L}}^{-1}\left[ \left( (1-\sigma )+\frac{\sigma }{s^{\sigma }}\right) {\textbf{L}}\left\{ \vartheta t^{\vartheta -1}(\omega _{2xxxxx}-\omega _{2xxx}-E_{2})\right\} \right] ,\\ \vdots . \end{aligned}$$

The whole series solution can be scripted as     $\omega (x,t)=\sum _{n=0}^\infty \omega _n(x,t)$.

## Validation of the proposed method

Here in this section we solve some of the examples by the proposed method discussed in “Existence of the initial value problems” section.

### *Example 1*

Consider Eq. (1) in Caputo sense which is given by

24 $$\begin{aligned} ^{C}D_t^{\sigma ,\,\vartheta }\omega -\frac{\partial ^{5} \omega }{\partial x^{5}}+\frac{\partial ^{3} \omega }{\partial x^{3}}+\omega \frac{\partial \omega }{\partial x}=0,\ \ \,0<\sigma ,\vartheta \le 1, \end{aligned}$$

with initial condition and exact solution is,

25 $$\begin{aligned} \omega (x,0)= & {} \rho +\eta sech^4(\nu x), \end{aligned}$$

26 $$\begin{aligned} \omega (x,t)= & {} \rho +\eta sech^4\nu \left( x+c t\right) . \end{aligned}$$

We find $\omega _0$, $\omega _1$, $\omega _2$ and so on in order to solve Eq. (24) .

Since we know that:

$$\begin{aligned} \omega (x,0)= & {} \rho +\eta sech^4(\nu x), \end{aligned}$$

after some calculation we get:

$$\begin{aligned} \omega _{1}= & {} \frac{4 \vartheta \eta \nu \Gamma (\vartheta ) t^{\sigma +\vartheta -1} \tanh (\nu x) \text {sech}^4(\nu x)}{\Gamma (\sigma +\vartheta )} \left( -256 \nu ^4+16 \nu ^2\right. \\{} & {} \left. +\rho +\left( -1680 \nu ^4+\eta \right) \text {sech}^4(\nu x)+30 \left( -\nu ^2+52 \nu ^4\right) \text {sech}^2(\nu x)+1\right) ,\\{} & {} \vdots . \end{aligned}$$

Thus, the solution is:

27 $$\begin{aligned} \omega (x,t)=\omega _0+\omega _{1}+\cdots . \end{aligned}$$

**Table 1:** Absolute value error.

(x,t)	Exact	$\omega$	$\mid$ Exact$-\omega \mid$	(x,t)	Exact	$\omega$	$\mid$ Exact$-\omega \mid$
(− 2,0.05)	0.1086	0.1086	1.4808 $\times \;10^{-06}$	(− 1,0.05)	0.1724	0.1724	2.3636 $\times \; 10^{-06}$
(0,0.05)	0.1953	0.1953	2.7108 $\times \; 10^{-06}$	(1,0.05)	0.1714	0.1714	2.3588 $\times \; 10^{-06}$
(2,0.05)	0.1068	0.1068	1.4734 $\times\; 10^{-06}$	(− 2,0.03)	0.1082	0.1082	5.3254 $\times \; 10^{-07}$
(− 1,0.03)	0.1722	0.1722	8.5054 $\times \;10^{-07}$	(0,0.03)	0.1953	0.1953	9.7589 $\times \; 10^{-07}$
(1,0.03)	0.1716	0.1716	8.4951 $\times \; 10^{-07}$	(2,0.03)	0.1072	0.1072	5.3096 $\times \;10^{-07}$

The above table shows the error in approximate vs Exact solution of the considered model with CFO for parameters taken as $\sigma =1,~\vartheta =1,~\nu =\frac{1}{2\sqrt{13}},~\rho =\frac{-72}{169},~ \eta =\frac{105}{169},~and~c=\frac{36}{169}$. From the Table 1, it is observable that the absolute error decreases as space variable *x* increases, at small time *t*.

**Figure 1:**
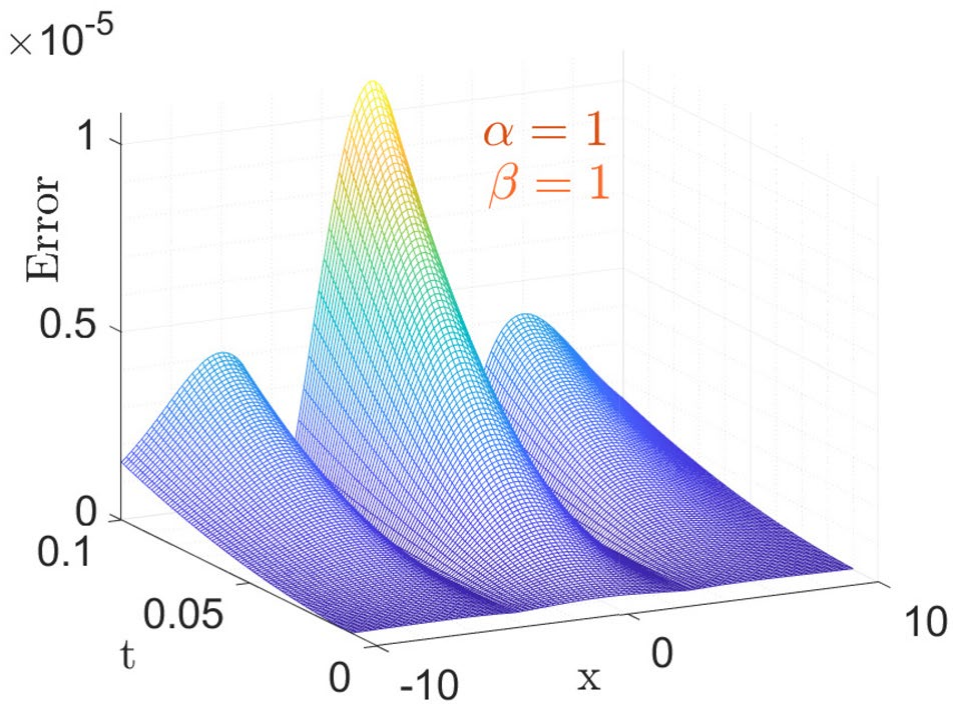
The surface plot of Error analysis for exact versus approximate with CFO.

Here, in Fig. 1 the parameters taken is same as above for the Table 1. Figure 1 shows the 3-dimensional graph of Error in Approximate solution of considered model with CFO. Anyone can get an Idea at a glance that how much the proposed method with CFO is efficient by giving such a negligible error vs Exact solution of considered model.

**Figure 2:**
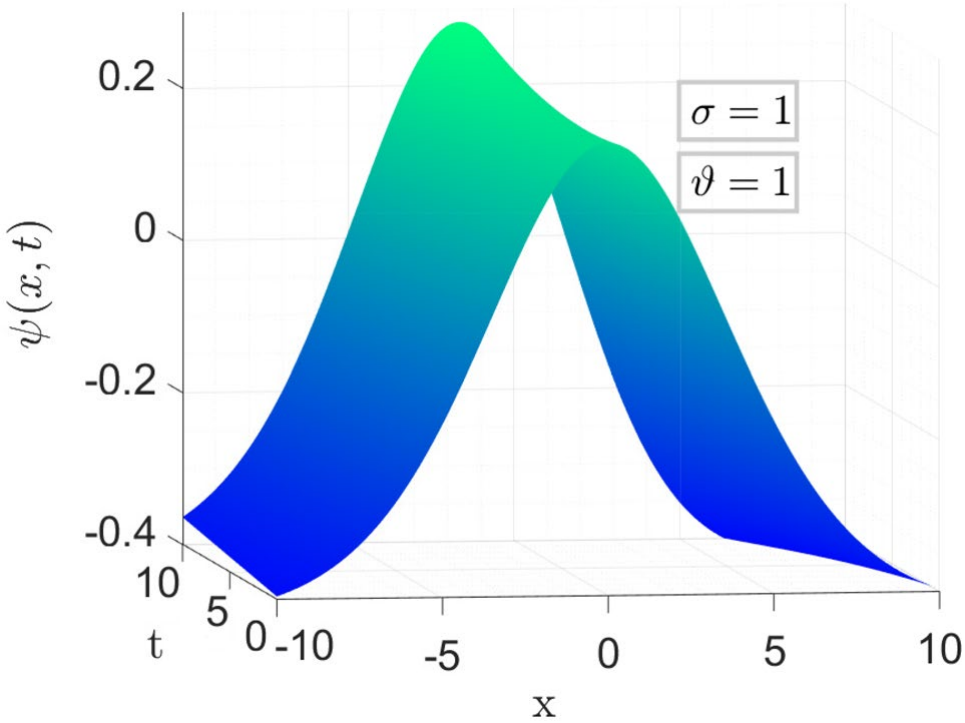
The surface plot of approximate solution Eq. (27).

Figure 2 is the 3d behavior of approximate solution (Eq. 27) for the parameters taken as same as above.

**Figure 3:**
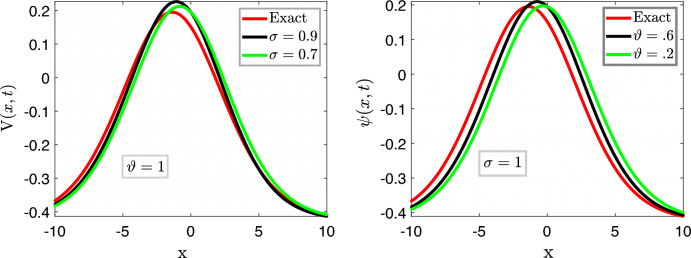
Comparison of Eqs. (26) vs (27) for different values of $\sigma$ and $\vartheta$ respectively.

In Fig. 3, we set the parameters as follows: $\nu =\frac{1}{2\sqrt{13}},~\rho =\frac{-72}{169},~ \eta =\frac{105}{169},~and~c=\frac{36}{169}$. The left plots depict a comparison between the Approximate solution Eq. (27) and the Exact solution Eq. (26) for different values of the fractional order variable, i.e., $\sigma =0.9$ and $\sigma =0.7$, while keeping the fractal variable $\vartheta$ fixed at 1, with a time variable $t=6.5$.

In the right plot, we illustrate the 2D behavior of the solution Eq. (27) of the model considered, in a fractal-fractional sense, comparing it with the exact solution Eq. (26) with integer order, for a fixed fractional variable $\sigma =1$ and varying fractal parameter $\vartheta =0.6,0.2$.

This analysis pertains to the fractal fractional Kawahara equation, which describes the evolution of wave phenomena in complex media with fractal and fractional characteristics.

We observe that for small values of *t*, the waves exhibit a close proximity to each other, indicating a subtle interplay between the fractional and fractal effects. Moreover, as time (*t*) increases, the system’s behavior becomes more pronounced, revealing intricate patterns and dynamics. Notably, we note a convergence of the approximate soliton solution towards the exact solution of the considered model, underscoring the robustness of the theoretical framework in capturing the underlying physics of the system.

**Figure 4:**
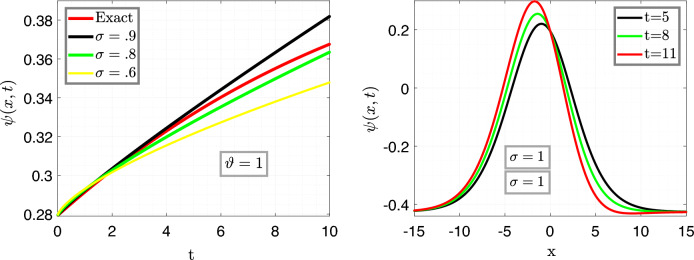
Time behavioral plots of considered model with CFO.

In Fig. 4, the left plot is the illustration of Eq. (27) vs time *t* for $x=6.5$ with varying fractional parameter $\sigma =0.9,~\sigma =0.8,~\sigma =0.6$ with fixed fractal variable $\vartheta =1$, moreover, the remaining parameters are taken same as above, and the right plot illustrate Eq. (27) for different value of time in order with fixed fractal and fractional variables $i-e$ $\sigma =1=\vartheta$.

### *Example 2*

Consider Eq. (1) with *CFFO*

28 $$\begin{aligned} ^{CF}D_t^{\sigma ,\,\vartheta }\omega -\frac{\partial ^{5} \omega }{\partial x^{5}}+\frac{\partial ^{3} \omega }{\partial x^{3}}+\omega \frac{\partial \omega }{\partial x}=0,\ \ \,0<\sigma \le 1, \end{aligned}$$

Equations (25) and (26) are the initial condition and exact solution, respectively. The following formula can be used to calculate Eq. (28) approximate solution:

$$\begin{aligned} \omega _{0}= & {} {\rho }+{\eta }sech^4(\nu x), \end{aligned}$$

after simplification we get:

$$\begin{aligned} \omega _{1}= & {} \frac{4 \eta \nu \vartheta t^{\vartheta -1} \tanh (\nu x) \text {sech}^4(\nu x) (\sigma t \Gamma (\vartheta )-(\sigma -1) \Gamma (\vartheta +1))}{\Gamma (\vartheta +1)}\left( \rho +\left( \eta -376 \nu ^4\right) \text {sech}^4(\nu x)\right. \\{} & {} \left. -256 \nu ^4 \tanh ^4(\nu x)+16 \nu ^2 \tanh ^2(\nu x)+2 \nu ^2 \left( 524 \nu ^2 \tanh ^2(\nu x)-7\right) \text {sech}^2(\nu x)+1\right) \\{} & {} \vdots . \end{aligned}$$

Same like above, we can also calculate $\omega _2$ and so on. The solution is describes as

29 $$\begin{aligned} \omega (x,t)=\omega _0+\omega _{1}+\cdots . \end{aligned}$$

.

**Table 2:** Error approximation.

(x,t)	Exact	$\omega$	$\mid$ Exact$-\omega \mid$	(x,t)	Exact	$\omega$	$\mid$ Exact$-\omega \mid$
(− 2,0.05)	0.1086	0.1046	4.0 $\times \; 10^{-3}$	(− 1,0.05)	0.1724	0.1701	2.3 $\times \; 10^{-3}$
(0,0.05)	0.1953	0.1953	2.7 $\times \;10^{-6}$	(1,0.05)	0.1714	0.1737	2.3 $\times \;10^{-3}$
(2,0.05)	0.1109	− 0.1109	4.0 $\times \; 10^{-3}$	(− 2,0.03)	0.1086	0.1046	4.0 $\times \;10^{-3}$
(− 1,0.03)	0.1724	0.1701	2.3 $\times \; 10^{-3}$	(0,0.03)	0.1953	0.1953	2.7 $\times \; 10^{-6}$
(1,0.03)	0.1714	0.1737	2.2 $\times \;10^{-3}$	(2,0.03)	0.1068	0.1109	4.0 $\times\; 10^{-3}$

The above table shows the error in approximate vs Exact solution of the considered model with CFO for parameters taken as $\sigma =1,~\vartheta =1,~\nu =\frac{1}{2\sqrt{13}},~\rho =\frac{-72}{169},~ \eta =\frac{105}{169},~and~c=\frac{36}{169}$. From the Table 2, it is observable that the absolute error decreases as space variable *x* increases, at small time *t*.

**Figure 5:**
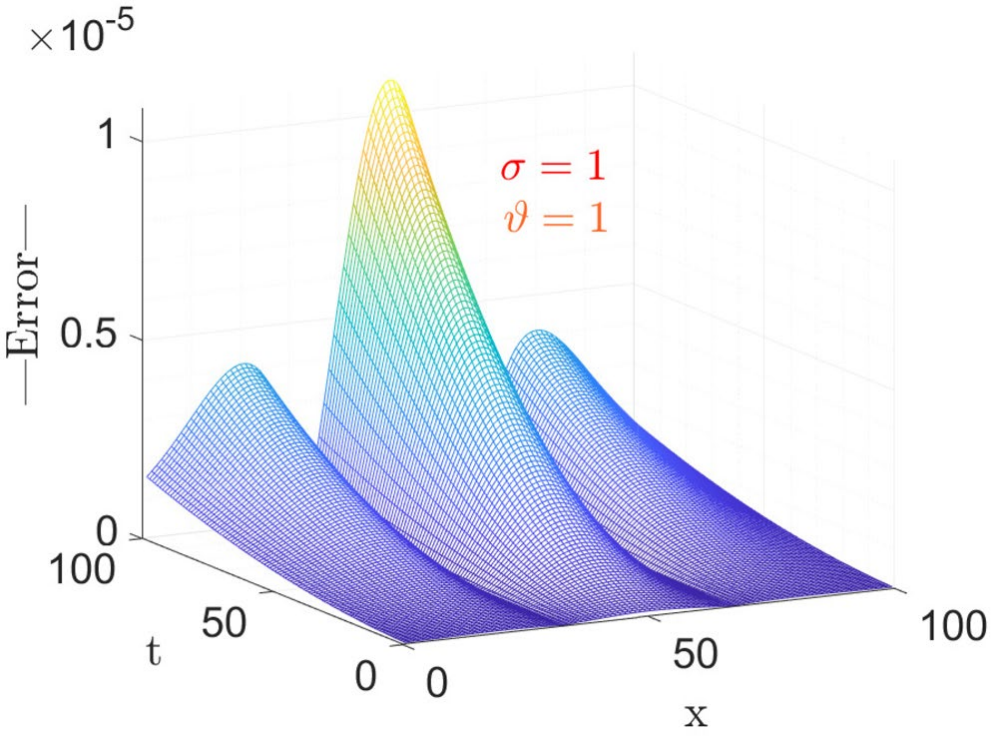
The surface plot of Error analysis for exact versus approximate with CFFO.

Here, in Fig. 1 the parameters taken is same as above for the Table 2. Figure 5 shows the 3-dimensional graph of Error in Approximate solution of considered model with CFO. Anyone can get an Idea at a glance that how much the proposed method with CFO is efficient by giving such a negligible error vs Exact solution of considered model.

**Figure 6:**
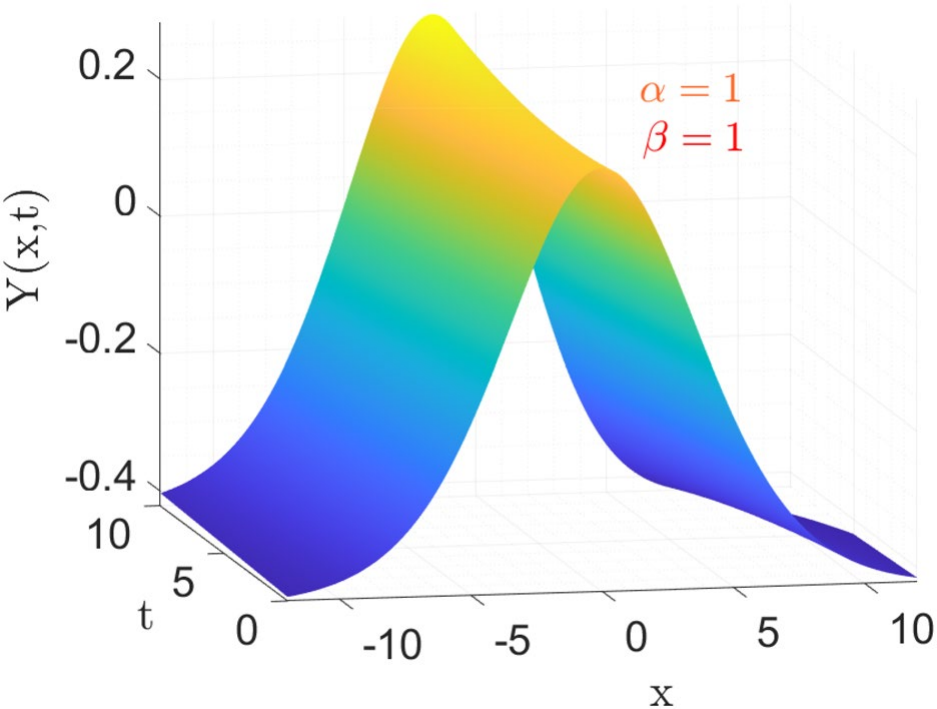
The surface plot of approximate solution Eq. (29).

Figure 6 is the 3d behavior of approximate solution (Eq. 29) for the parameters taken as same as above.

**Figure 7:**
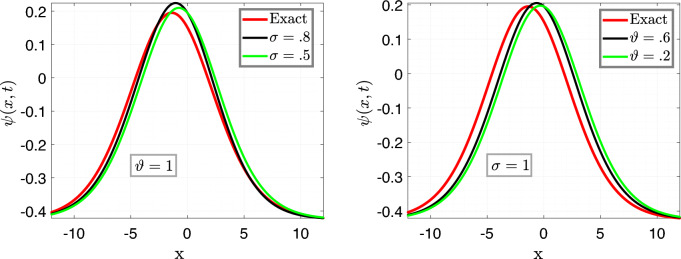
Comparison of Eqs. (29) vs (26) for different values of $\sigma$ and $\vartheta$ respectively.

We explore the dynamics of the Caputo-Fabrizio fractal fractional operator in the framework of the Kawahara equation in Fig. 7. $\nu =\frac{1}{2\sqrt{13}},\rho =\frac{-72}{169},\eta =\frac{105}{169}, andc=\frac{36}{169}$ are the parameters that have been specified.

The left plots provide a comparison of the different fractional order variables ($\sigma =0.9$ and $\sigma =0.7$) between the approximate solution Eq. (29) and the exact solution Eq. (26). The time variable stays at $t=6.5$, while the fractal variable is held constant at $\vartheta =1$.

As for the right figure, it explores the 2D behavior of the precise solution Eq. (26) under integer order in comparison to the solution Eq. (29) under the Caputo-Fabrizio fractal fractional operator. In this case, the fractal parameter ($\vartheta =0.6,0.2$) is varied while the fractional variable is fixed at $\sigma =1$. These studies illuminate wave processes in complicated media, capturing fractional and fractal properties in the context of the Kawahara equation. After investigation, we find that the waves show an impressive coherence at tiny time steps, suggesting complex interactions between fractional and fractal constituents. The system’s behavior becomes increasingly apparent with time, exposing complex dynamics and changing patterns. We see that the approximation soliton solution converges noticeably to the precise solution, demonstrating the ability of the theoretical framework to accurately describe the physics underlying the system.

**Figure 8:**
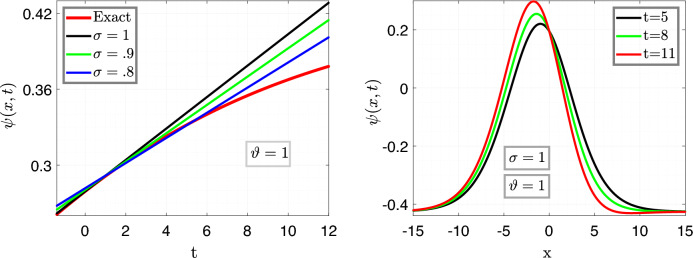
Time behavioral plots of considered model with CFFO.

In Fig. 8, the left plot is the illustration of Eq. (29) vs time *t* for $x=6.5$ with varying fractional parameter $\sigma =1.0,~\sigma =0.9,~\sigma =0.8$ with fixed fractal variable $\vartheta =1$, moreover, the remaining parameters are taken same as above, and the right plot illustrate Eq. (29) for different value of time in order with fixed fractal and fractional variables $i-e$ $\sigma =1=\vartheta$.

### *Example 3*

Consider Eq. (1) with Mittag Leffler kernel

30 $$\begin{aligned} ^{ABC}D_t^{\sigma ,\,\vartheta }\omega -\frac{\partial ^{5} \omega }{\partial x^{5}}+\frac{\partial ^{3} \omega }{\partial x^{3}}+\omega \frac{\partial \omega }{\partial x}=0,\ \ \,0<\sigma \le 1 \end{aligned}$$

Equations (25) and (26) are the initial condition and exact solution, respectively. The following formula can be used to calculate Eq. (26) approximate solution:

$$\begin{aligned} \omega _{0}= & {} {\rho }+{\eta }sech^4(\nu x), \\ \omega _{1}= & {} \frac{4 \eta \nu \vartheta t^{\vartheta -1} \tanh (\nu x) \text {sech}^4(\nu x) \left( (1-\sigma ) \Gamma (\sigma +\vartheta )+\sigma \Gamma (\vartheta ) t^{\sigma }\right) }{\Gamma (\sigma +\vartheta )} \left( -256 \nu ^4+16 \nu ^2\right. \\{} & {} \left. +\rho +\left( -1680 \nu ^4+\eta \right) \text {sech}^4(\nu x)+\left( 1560 \nu ^4-30\nu ^2\right) \text {sech}^2(\nu x)\right) , \\ \vdots . \end{aligned}$$

Same like above we can find other terms. The solution is describes as:

31 $$\begin{aligned} \omega (x,t)=\omega _0+\omega _{1}+\mathcal {O}(1)+\cdots . \end{aligned}$$

.

**Table 3:** Error approximation.

(x,t)	Exact	$\omega$	$\mid$ Exact$-\omega \mid$	(x,t)	Exact	$\omega$	$\mid$ Exact$-\omega \mid$
(− 2,0.2)	0.1111	0.1111	2.3867 $\times \; 10^{-05}$	(− 1,0.2)	0.1738	0.1738	3.7930 $\times\; 10^{-05}$
(0,0.2)	0.1952	0.1953	4.3371 $\times \; 10^{-05}$	(1,0.2)	0.1699	0.1700	3.7626 $\times \; 10^{-05}$
(2,0.2)	0.1043	0.1043	2.3309 $\times\; 10^{-05}$	(− 2,0.4)	0.1144	0.1145	9.6402 $\times\; 10^{-05}$
(− 1,0.4)	0.1756	0.1758	1.5231 $\times \;10^{-04}$	(0,0.4)	0.1951	0.1953	1.7346 $\times \; 10^{-04}$
(1,0.4)	0.1679	0.1680	1.4988 $\times \; 10^{-04}$	(2,0.4)	0.1008	0.1009	9.2654 $\times \; 10^{-05}$

The above table shows the error in approximate vs Exact solution of the considered model with ABC fractional operator for parameters taken as $\sigma =1,~\vartheta =1,~\nu =\frac{1}{2\sqrt{13}},~\rho =\frac{-72}{169},~ \eta =\frac{105}{169},~and~c=\frac{36}{169}$. From the Table 3, it is observable that the absolute error decreases as space variable *x* increases, at small time *t*.

**Figure 9:**
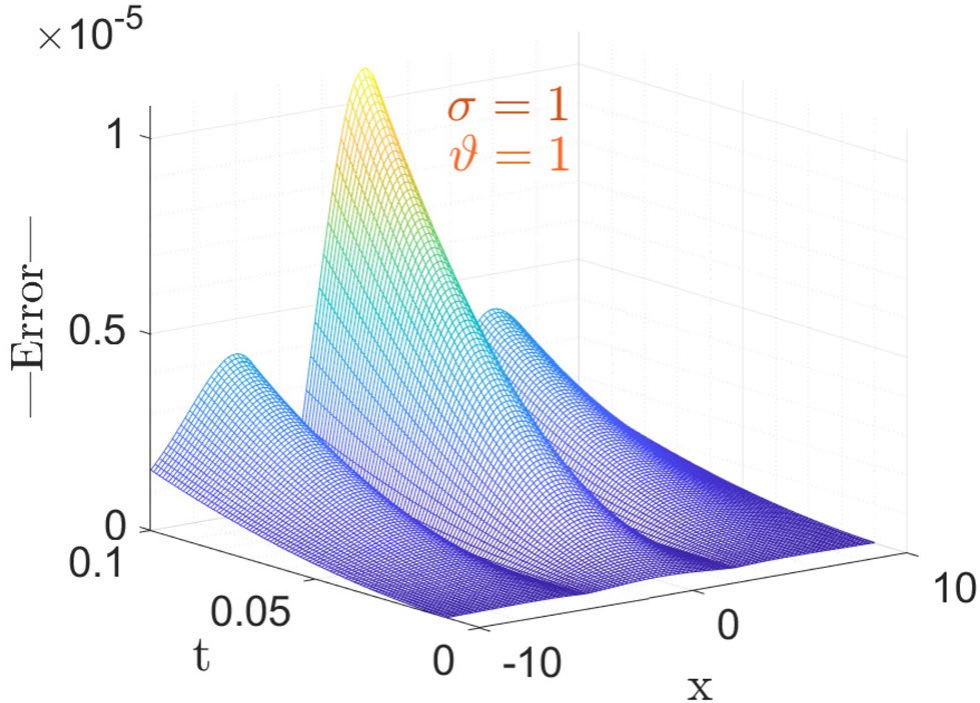
The surface plot of Error analysis between exact and approximate with ABC fractional operator.

Here, in Fig. 9 the parameters taken is same as above for the Table 3. Figure 9 shows the 3-dimensional graph of Error in Approximate solution of considered model with ABC. Anyone can get an Idea at a glance that how much the proposed method with ABC fractional operator is efficient by giving such a negligible error vs Exact solution of considered model.

**Figure 10:**
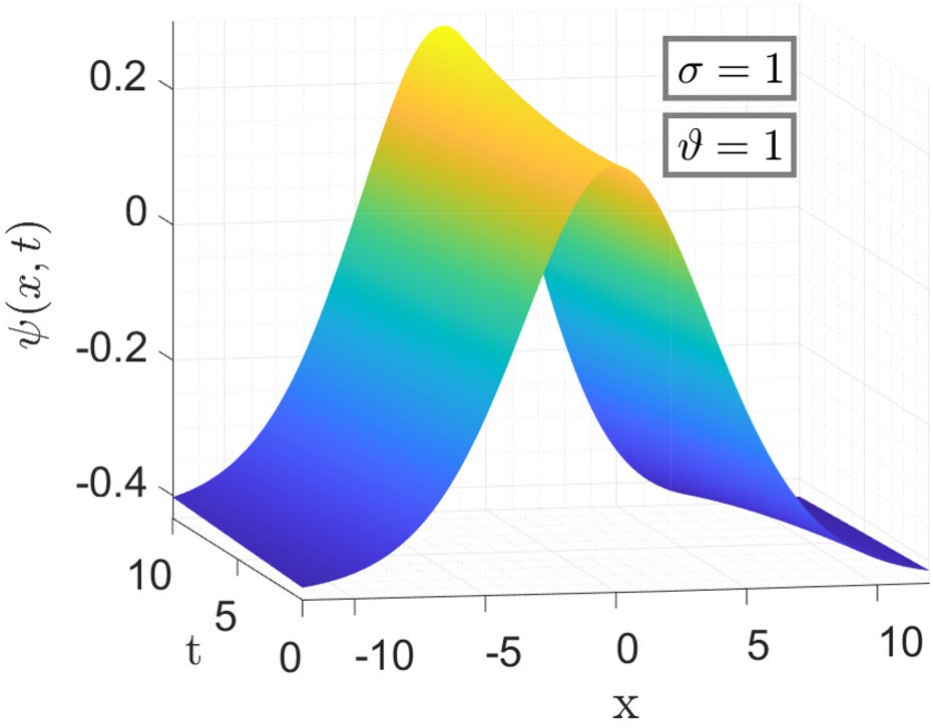
The surface plot of approximate solution Eq. (31).

Figure 10 is the 3d behavior of approximate solution (Eq. 31) for the parameters taken as same as above.

**Figure 11:**
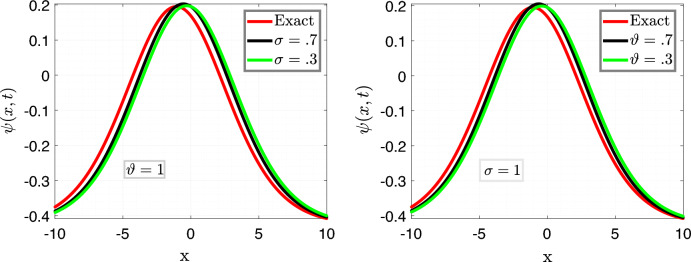
Comparison of Eqs. (26) and (31) for different values of $\sigma$ and $\vartheta$ respectively.

We investigate the fractal fractional Kawahara equation dynamics using the ABC fractal fractional operator, as shown in Fig. 11. The parameters that were selected are as follows: $\nu =\frac{1}{2\sqrt{13}}$, $\rho =\frac{-72}{169}$, $\eta =\frac{105}{169}$, and $c=\frac{36}{169}$. The figure’s left plots provide a comparison of the equation’s approximate Eq. (31) and exact Eq. (26) solutions. We examine how the fractional order variable $\sigma$ behaves at various values, namely $\sigma =0.7$ and $\sigma =0.3$, while maintaining the constant value of the fractal variable $\vartheta$ at $\vartheta =1$. The temporal evolution is fixed at $t=5$ in this case. However, when compared to the integer-order exact solution Eq. (26), the right plot reveals the complex 2D trajectory of the solution presented in Eq. (31). The fractal parameter $\vartheta$ assumes different values: $\vartheta =0.7$ and $\vartheta =0.3$, while the fractional variable $\sigma$ stays constant at $\sigma =1$. During our investigation, we find remarkable physical insights. First, at shorter time intervals, the waves show a remarkable closeness, highlighting the interaction of the system’s aspects. Furthermore, the system exhibits a measurable progression throughout time, with each instant enhancing its dynamic diversity. Especially, we see a strong convergence: over time, the approximation soliton solution smoothly approaches the precise solution, demonstrating the stability of our model and its ability to describe real-world processes.

**Figure 12:**
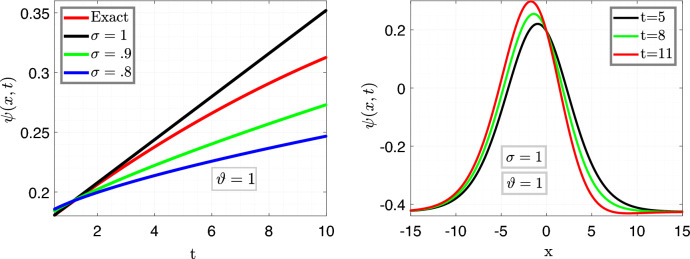
Time behavioral plots of considered model with ABC fractional operator.

In Fig. 12, the left plot is the illustration of Eq. (31) vs time *t* for $x=5$ with varying fractional parameter $\sigma =1.0,~\sigma =0.9,~\sigma =0.8$ with fixed fractal variable $\vartheta =1$, moreover, the remaining parameters are taken same as above, and the right plot illustrate Eq. (31) for different value of time in order with fixed fractal and fractional variables $i-e$ $\sigma =1=\vartheta$.

## Comparative analysis

Here in this section we shows the results obtained on all of the above mentioned operator.

**Table 4:** Comparison between Caputo fractal fractional, Caputo-Fabrizio, ABC operator.

(x,t)	Exact	Caputo	$\mid Exact-Caputo \mid$	CF	$\mid Exact-CF \mid$	ABC	$\mid Exact-ABC \mid$
(− 3.0,0.05)	0.0187	0.0205	− 1.8 $\times \; 10^{-03}$	0.0246	− 5.9 $\times \; 10^{-03}$	0.0258	− 7.1 $\times 10^{-4}$
(− 2.5,0.05)	0.0658	0.0674	− 1.6 $\times\; 10^{-03}$	0.0713	− 5.5$\times\; 10^{-03}$	0.0724	− 6.7 $\times \; 10^{-04}$
(− 2.0,0.05)	0.1086	0.1100	− 1.5 $\times\; 10^{-03}$	0.1134	− 4.9 $\times \;10^{-03}$	0.1144	− 5.9 $\times \; 10^{-04}$
(− 1.5,0.05)	0.1448	0.1460	− 1.2 $\times\; 10^{-03}$	0.1487	− 3.9 $\times\; 10^{-03}$	0.1496	− 4.8 $\times \; 10^{-04}$
(− 1.0,0.05)	0.1724	0.1732	− 8.3 $\times \;10^{-04}$	0.1752	− 2.8 $\times \;10^{-04}$	0.1757	− 3.4 $\times \;10^{-04}$
(− 0.5,0.05)	0.1896	0.1900	− 4.3 $\times\; 10^{-04}$	0.1910	− 1.4 $\times \;10^{-04}$	0.1913	− 1.7 $\times \; 10^{-04}$
(0.0,0.05)	0.1953	0.1953	− 2.7 $\times \; 10^{-06}$	0.1953	− 2.7 $\times \; 10^{-06}$	0.1953	− 2.7 $\times \; 10^{-06}$
(0.5,0.03)	0.1892	0.1888	3.2 $\times \; 10^{-04}$	0.1877	1.5 $\times \; 10^{-04}$	0.1875	1.7 $\times\; 10^{-04}$
(1.0,0.03)	0.1716	0.1710	6.2 $\times\; 10^{-04}$	0.1688	2.8 $\times \; 10^{-04}$	0.1683	3.3 $\times \; 10^{-04}$
(1.5,0.03)	0.1437	0.1428	8.9 $\times \;10^{-04}$	0.1397	4.0 $\times \;10^{-04}$	0.1391	4.6 $\times\; 10^{-04}$
(2.0,0.03)	0.1072	0.1061	1.1 $\times \; 10^{-04}$	0.1022	5.0 $\times \; 10^{-04}$	0.1015	5.7 $\times\; 10^{-04}$
(2.5,0.03)	0.0642	0.0630	1.2 $\times \; 10^{-03}$	0.0586	5.6 $\times \; 10^{-03}$	0.0577	6.5 $\times \; 10^{-04}$
(3.0,0.03)	0.0171	0.0157	1.3 $\times \; 10^{-03}$	0.0111	6.0 $\times \; 10^{-03}$	0.0101	6.9 $\times \; 10^{-04}$

**Figure 13:**
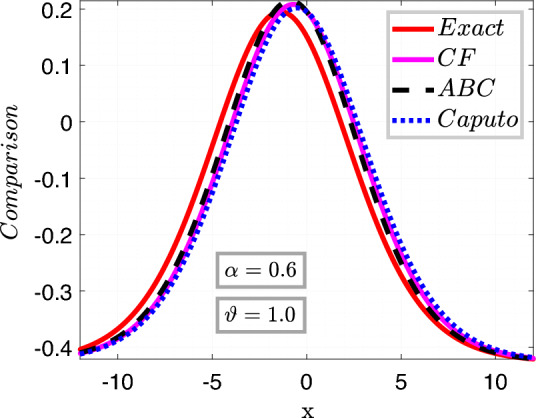
Comparison analysis of different fractional order.

While fractal fractional operators such as the Caputo, Caputo Fabrizio, and ABC operators are scrutinized by means of Laplace Adomian decomposition techniques, a comparison of the outcomes displays in Table 4 and graphically represented in Fig. 13, we observe that the ABC operator accomplishes better than the others. Even though all operators are convenient for simulating sophisticated systems with fractional derivatives, the ABC operator is more accurate and efficient in capturing the complex behavior of fractal events. The ABC operator’s supremacy arises from its exceptional capacity to provide a more adaptable framework for explaining anomalies and non-local behaviors seen in fractal systems. In contrast to the Caputo and Caputo Fabrizio operators, the ABC operator adds a new parameter that allows for more precise modifications to the fractional order, improving its flexibility to adapt to a wider range of fractal concepts. As a result, the ABC operator is the go-to option for both researchers and practitioners in various domains, including engineering and the natural sciences, since it produces more accurate findings and makes it easier to comprehend the underlying dynamics of fractal systems.

### Ethical declaration

In this study, human data has not been used for modeling.

## Conclusion

This research study has given a way for solving the Kawahara equation using the Laplace transform Adomian decomposition method (LADM) under three different fractal fractional differential operators: Caputo, Caputo-Fabrizio, and ABC. We have proved the solution’s existence and uniqueness of solution via advanced fixed point theorems. Our results show that the suggested approach offers an effective and precise solution for nonlinear partial differential equations like the Kawahara problem which has been observed in absolute error in the tables. A comparison between results under three different have been presented via tables and graphs to figure out that ABC operator provide good results due to nonsingular and nonlocal kernel. Contributions of this paper include a new approach to partial differential equations solution based on fractal fractional differential operators and the LADM. The LADM can be used to other nonlinear issues in physics, engineering, and other disciplines. It also has the benefit of offering effective numerical solutions that work for a variety of issues. We hope that our results will stimulate additional investigation into the application of fractal fractional differential operators and the LTADM for resolving complex issues and promote the creation of more effective and precise numerical methods for solving partial differential equations. By means of our investigation, we provide a valuable contribution to the rapidly developing domain of fractional calculus and establish a foundation for forthcoming research paths that aim to fully use fractal fractional approaches in the modeling of intricate physical processes.

Now a days delay differential equations, neural network approach and fractional calculus have many applications in the different fields of sciences such as bifurcation and BAM neural nework^37,38^, predator-prey and Lotka-Volterra system^39,40^, plankton-oxygen model^41^, and others^42,43^. Using these approaches, one can find soliton solutions for the considered system.

## Data Availability

The datasets generated during the current study are available from the corresponding author (Mati ur Rahman) on reasonable request.
